# Exploring the importance of cancer pathways by meta-analysis of differential protein expression networks in three different cancers

**DOI:** 10.1186/s13062-016-0168-8

**Published:** 2016-12-20

**Authors:** Sinjini Sikdar, Somnath Datta, Susmita Datta

**Affiliations:** Department of Biostatistics, University of Florida, Gainesville, FL 32611 USA

**Keywords:** ICGC, Signaling pathway, Network, Protein, Cancer

## Abstract

**Background:**

It is believed that all cancers occur due to the mutation or change in one or more genes. In order to investigate the significance of the biological pathways which are interrupted by these genetic mutations, we pursue an integrated analysis using multiple cancer datasets released by the International Cancer Genome Consortium (ICGC). This dataset consists of expression profiles for genes/proteins of patients receiving treatment, for three types of cancer - Head and Neck Squamous Cell Carcinoma (HNSC), Lung Adenocarcinoma (LUAD) and Kidney Renal Clear Cell Carcinoma (KIRC). We consider pathway analysis to identify all the biological pathways which are active among the patients and investigate the roles of the significant pathways using a differential network analysis of the protein expression datasets for the three cancers separately. We then integrate the pathway based results of all the three cancers which provide a more comprehensive picture of the three cancers.

**Results:**

From our analysis of the protein expression data, overall, RAS and PI3K signaling pathways appear to play the most significant roles in the three cancers - Head and Neck Squamous Cell Carcinoma (HNSC), Lung Adenocarcinoma (LUAD) and Kidney Renal Clear Cell Carcinoma (KIRC).

**Conclusion:**

This analysis suggests that the RAS and PI3K signaling pathways are the two most important pathways in all the three cancers and should be investigated further for their potential roles in cancers.

**Reviewers:**

This article was reviewed by Joaquin Dopazo and Samiran Ghosh.

## Background

Several studies have found that there are approximately 140 “driver” genes which can promote the formation of tumors if affected by intragenic mutations. These “driver genes” are known to be directly or indirectly responsible for causing selective growth advantage. These “driver genes” are classified into twelve signaling pathways. This selective growth advantage can occur only through these twelve signaling pathways [1]. We refer these twelve signaling pathways as “target pathways”. It is also found that the “target pathways” regulate three core cellular processes - “cell fate”, “cell survival” and “genome maintenance” [1] . We believe understanding the roles of these twelve pathways can result into novel therapeutic intervention strategy. In this paper, we undertake a novel investigation of the roles of these “target pathways” using a differential network analysis of the protein expression datasets on three cancers namely, Head and Neck Squamous Cell Carcinoma (HNSC), Lung Adenocarcinoma (LUAD) and Kidney Renal Clear Cell Carcinoma (KIRC). We find the study of protein expressions is justified as the mutations of the previously mentioned “driver genes” alter the protein products. These datasets are available to us from International Cancer Genomic Consortium (ICGC) as part of the CAMDA 2015 challenge data. We pursue an integrative analysis of protein expressions of all these three cancer datasets to investigate whether each of these “target pathways” plays a significant role in these three cancers. For example, we determine whether the proteins associated with these pathways interact differently between the two clinical groups (“progression” or “complete remission”) of patients. The differentially expressed pathways between the two disease groups allow us to gain more insights about the functional working mechanism of the cells than just the individual differentially expressed genes/proteins [2]. The process begins with grouping of the proteins according to their biological pathways, as described in the section- *Pathway analysis*. Then, in the Section- *Differential network analysis*, we examine whether the network structures of all the proteins in these “target pathways” have changed from the “complete remission” group of patients to the “progression” group. We also examine whether the connectivity of each single protein in the networks of proteins in these “target pathways” has changed between the two groups. Then we rank the “target pathways” as well as the constituent proteins to get overall ranked lists which would then rank the pathways by their global order of importance with respect to all the three cancers (section- *Rank aggregation*). This ranking may shed light to the regulatory roles of individual proteins in the context of all others in the pathway. The rest of the article is organized as follows. In Results section, we report the results of each of the analysis. Then, we conclude with a Discussion section. Then in Methods section, we describe the datasets used and all the methods used for the analysis.

## Results

### Pathway analysis results

For each cancer type, we find representation of five out of twelve “target pathways” in our sample of 132 proteins using “GO” clustering tool [3, 4]; they are the PI3K signaling pathway, Cell Cycle, Apoptosis, RAS signaling pathway and MAPK signaling pathway. It is interesting to note that all these five “target pathways” are all related to “cell survival” function.

### Differential network analysis results

Next, we determine whether there is any significant difference in the network structures of the “target pathways” between the two groups of patients (“progression” vs “complete remission”). We perform differential network analysis [5, 6] of the network of proteins in these representative “target pathways” between the two groups of patients (“progression” vs “complete remission”) using the test statistic given in Equation 1, with Pearson’s correlation coefficients as connectivity scores and absolute distance measure for each of the three cancer types. The p-values of the analysis are reported in Table 1. Based on these results, we have the following findings: the RAS signaling pathway is highly significant (*p*-value = 0.026) and MAPK signaling pathway is marginally significant (*p*-value = 0.082) in HNSC; for LUAD, PI3K signaling pathway is highly significant (*p*-value = 0.013).

**Table 1:** “Target pathways” along with the p-values obtained from differential network analysis for each cancer type

Target Pathway	Cancer Type		
	HNSC	LUAD	KIRC
RAS signaling pathway	0.026	0.507	0.156
MAPK signaling pathway	0.082	0.759	0.517
PI3K signaling pathway	0.241	0.013	0.774
Apoptosis	0.407	0.417	0.487
Cell Cycle	0.410	0.238	0.997

**Fig. 1:**
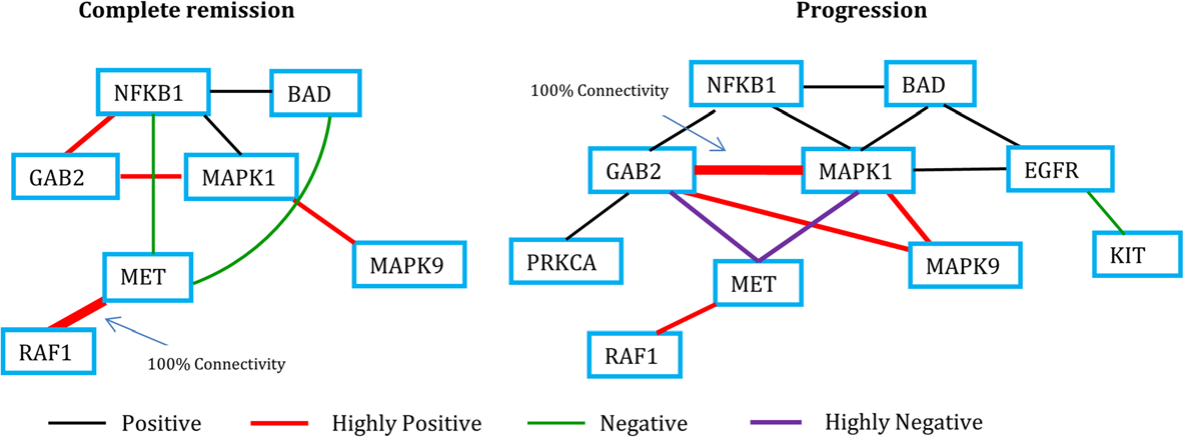
Network structure for RAS signaling pathway in Head and Neck Squamous Cell Carcinoma (HNSC)

A graphical representation of the network structure of the proteins in the two groups of patients for RAS signaling pathway in HNSC is shown in Fig. 1. In this figure, two proteins are connected if the connectivity score between them is significantly large. Different colors and shades in the figure represent positive or negative correlations and the thickness of the lines represents the strength of the associations. A visual inspection reveals some obvious differences in the network connectivity between the two groups of patients. It can be seen from Fig. 1 that the protein MET has very high connectivity with the proteins GAB2 and MAPK1 in the “progression” group, whereas no such connectivity can be seen between them in the “complete remission” group. On the other hand, MET can be seen to be connected with NFKB1 and BAD in the “complete remission” group but no such connectivity can be seen in the “progression” group. Also, it is interesting to note that MET has 100% connectivity with RAF1 in the “complete remission” group. The protein GAB2 appears to be highly connected with MAPK9 in the “progression” group but no connectivity can be seen between them in the “complete remission” group. GAB2 is also connected with PRKCA only in the “progression” group. Further, GAB2 has 100% connectivity with MAPK1 in the “progression” group. In the “progression” group, MAPK1 and BAD are connected among themselves and also with EGFR, which is further connected with KIT. But no such interesting connections can be seen in the “complete remission” group. Summarizing, GAB2, MAPK1, MET, and BAD show noticeably different connectivities in the two networks. The corresponding genes are known oncogenes. GAB2 is known to be overexpressed in multiple human tumors especially in melanoma. It is altered by MAPK and PI3K signaling pathways [7]. MAPK1 (Mitogen-activated protein kinase 1) is broadly implicated in many cancers [8]. MET is associated with MET signaling process. In many cancers involving solid tumors, inhibiting this signaling has major therapeutic effect [9]. Similarly, it is found that BAD is a pro-apoptotic protein which has been identified as an integrator of several anti-apoptotic signaling pathways in prostate cancer cells [10].

**Fig. 2:**
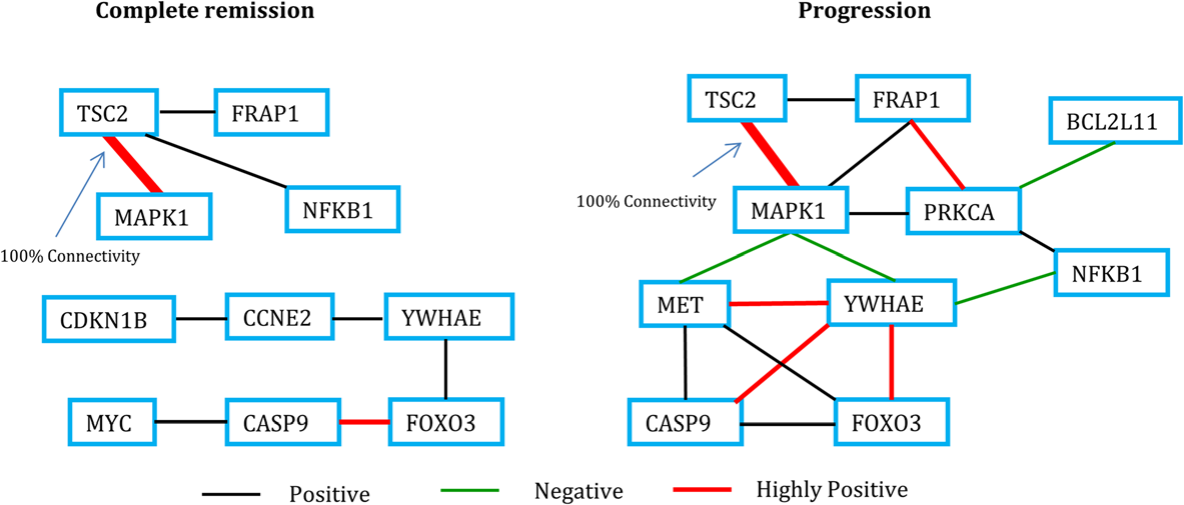
Network structure for PI3K signaling pathway in Lung Adenocarcinoma (LUAD)

A graphical representation of the network structure of the proteins in PI3K signaling pathway for the two groups of patients in LUAD is shown in Fig. 2. From the figure, it can be seen that the protein MET has interesting connections with the proteins CASP9, FOXO3, YWHAE and MAPK1 in the “progression” group whereas no such interesting connections can be found in the “complete remission” group. Also, PRKCA is connected with FRAP1, MAPK1, BCL2L11 and NFKB1 in the “progression” group but no such connectivity can be seen in the “complete remission” group. The protein MAPK1 seems to be connected with MET, YWHAE, PRKCA, FRAP1 and TSC2 in the “progression” group whereas it is only connected with TSC2 in the “complete remission” group. Here, MET, PRKCA and MAPK1 show significant differences in the two networks. PRKCA is a serine/threonine-protein kinase that is highly expressed in a number of cancer cells where it can act as a tumor promoter and is implicated in malignant phenotypes of several tumors such as gliomas and breast cancers [11].

**Fig. 3:**
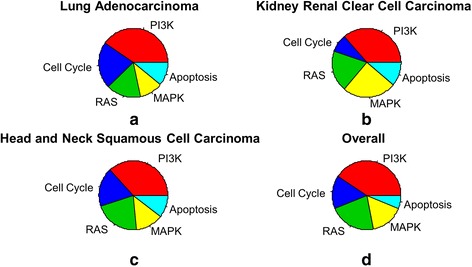
Relative contributions of the “target pathways” in the three cancers separately as well as combined

Our analysis of individual proteins using the test statistic given in Equation 4 produces Fig. 3 (a-c). The pie charts represent the proportions of top fifty differentially connected proteins for each of the “target pathways” in the three cancers. This provides a global visual representation of the relative importance of the “target pathways” in all three cancers. For all the three cancers, PI3K and RAS signaling pathways show significant contributions in terms of differential network connectivity.

### Rank aggregation results

Next, we rank the relative importance of the “target pathways” based on the p-values, obtained using Equation 3 from the differential network analysis, so that we can get an idea about the ordering of importance of the “target pathways”. Since, the ordering vary from one cancer to another; we obtain a rank aggregated list of the “target pathways” using *RankAggreg* [12, 13]. Table 2 shows the ordering of the five “target pathways” for the three cancers along with the rank aggregated list. Thus overall, the RAS signaling pathway appears to be most important followed by the PI3K signaling pathway, based on our integrative analysis of the available data on three cancers.

**Table 2:** “Target pathways” ordered by *p*-values for each cancer type along with the overall ordering

Cancer type	Pathway ordering by *p*-values
HNSC	R, M, P, A, C
LUAD	P, C, A, R, M
KIRC	R, A, M, P, C
Overall	R, P, M, A, C

*R* RAS Signaling pathway, *M* MAPK Signaling pathway, *P* PI3K Signaling pathway

*A* Apoptosis, *C* Cell Cycle

We also obtain a rank aggregated list of the proteins, based on the p-values of the tests based on differential connectivity of each protein in the two different networks of the two patient groups. The proportions of top fifty differentially connected proteins for each of the “target pathways” are shown in Fig. 3(d). Once again, PI3K and RAS take the top two most important spots in terms of differential network connectivity.

## Discussion and Conclusion

It is known that for most cancers with solid tumors the genes in the above mentioned “target pathways” display somatic mutations and change their protein products [1] . In all human tumors, PI3K is known to be as one of the most frequently targeted pathways. Mutation in PI3K pathway components contributes up to 30% of all human cancer and is known to be activated by RAS [14]. It is interesting to know that PI3K is a regulatory subunit, which binds to cell-surface receptors and to the RAS protein. Genes and proteins in PI3K and RAS have been investigated as therapeutic targets for many cancers [15, 16]. Here this purely quantitative analysis of the existing protein expression data of three different cancers also reveals the significant alteration of the proteins in PI3K and RAS pathways. Our findings are consistent with this and suggest that continued future efforts be made in this direction.

Since genes act in consort during a biological process, a network analysis is essential for a system-wide understanding. Thus, a study of differential network connectivity could yield interesting findings that are not possible from a differential expression analysis of individual proteins. In addition, pathway level information should be incorporated in a differential network analysis whenever possible. It can be seen from Fig. 3(d) that on the basis of analysis of differential connectivity of individual proteins, PI3K emerged as the most important pathway overall. However, this could be partly due to the fact that PI3K pathway has the largest number of proteins compared to the other “target pathways”. On the other hand, the test statistic based on differential connectivity of pathways is automatically normalized by the size of the pathway and on the basis of this comparison, RAS turned out to be the most significant overall as shown in Table 2.

In a recent study [17], multiple molecular profile data of LUAD for the CAMDA 2014 lung adenocarcinoma challenge data provided by ICGC is analyzed and it is noted that EGFR signaling pathway plays a significant role among the patients. Besides, it is known that EGFR activation initiates RAS signaling [18–20], and EGFR induces rapid increase in number of epithelial cells by activating a network of signaling elements, including members of the RAS and PI3K [20, 21]. Thus, it is common for lung cancer patients to have active EGFR mutation. Moreover, the RTK/RAS/RAF pathway, identified as the main route in causing adenocarcinoma, is shown to be activated in patients with “common” pathway mutations, e.g., in KRAS, BRAF, and EGFR [22]. Although the EGFR kinase inhibitor Gefitinib is an effective treatment for lung cancers with EGFR activating mutations, amplification of MET causes Gefitinib resistance by driving ERBB3 (HER3)–dependent activation of PI3K [23], a pathway thought to be specific to EGFR/ERBB family receptors. This fact, along with the fact that MET has been found in the module of the “progression” group but not in that of the “complete remission” group in PI3K signaling pathway of LUAD (see Fig. 2) in our current study, suggest that the patients under study might have been treated with Gefitinib, but the presence of MET in some of these patients (those in the “progression” group) may have led to the resistance to this drug. However, this is subject to verification as we don’t have the information regarding the treatment regime for any of these patients. This highlights the potential role of MET in lung cancer progression.

An interesting observation from Fig. 3 is that much lesser proportion of proteins in cell cycle pathway is differentially connected between the two groups of patients in KIRC compared to LUAD and HNSC. So underlying molecular mechanisms related to cell cycle pathway may be a little different in KIRC compared to LUAD and HNSC.

In a recent paper [24], patient level information such as mutation profiles is incorporated to identify protein-protein interaction (PPI) interfaces enriched in somatic mutations. It will be interesting to explore how to incorporate patient heterogeneity information into our approach.

## Methods

We have analyzed the preprocessed challenge datasets for CAMDA 2015 provided by the International Cancer Genomic Consortium (ICGC). For our study, we have considered the protein expression and the clinical profiles of the patients for the three cancers: Head and Neck Squamous Cell Carcinoma (HNSC), Lung Adenocarcinoma (LUAD), and Kidney Renal Clear Cell Carcinoma (KIRC). A set of 132 proteins is present in the protein expression profiles of each of the three cancers; the patient sample sizes of HNSC, LUAD and KIRC are 212, 237 and 454 patients, respectively. The number of patients in the clinical profile of HNSC, LUAD and KIRC are respectively 422, 473 and 515. The clinical profile of each of the cancer type represents the disease status (“progression” or “complete remission”) of each patient. However, 44, 111 and 28 patients have missing disease status in the clinical data of HNSC, LUAD and KIRC respectively. After removing the patients who have missing disease status, we are left with 185 patients (71 in “progression” group and 114 in “complete remission” group) in HNSC, 172 patients (51 in “progression” group and 121 in “complete remission” group) in LUAD and 430 patients (132 in “progression” group and 298 in “complete remission” group) in KIRC, each with expression values of 132 proteins. In summary, we have two groups of patients for each cancer type and a set of recorded protein expression values of 132 proteins for each of them.

### Pathway analysis

From several studies, it has been found that approximately there are 140 genes that are responsible for selective growth advantage. These genes are termed as “driver” genes. As mentioned before in the *Background*, mutations occur in a typical tumor due to two to eight such “driver” genes, while there are only twelve signaling pathways which allow selective growth advantage. These twelve signaling pathways (“target pathways”) are: $\mathrm{T}\mathrm{G}\mathrm{F} - \upbeta$, MAPK, STAT, PI3K, RAS, Cell Cycle/Apoptosis, NOTCH, HH, APC, Chromatin modification, Transcriptional regulation and DNA damage control. Among these, $\mathrm{T}\mathrm{G}\mathrm{F}-\upbeta$, MAPK, STAT, PI3K, RAS and Cell Cycle/Apoptosis regulate “cell survival”; NOTCH, HH, APC, Chromatin modification and Transcriptional regulation regulate “cell fate”; while the DNA damage control signaling pathway regulates “genome maintenance”. We have separately analyzed the protein profiles of the three cancer types using “GO” clustering tool [3, 4] and grouped the proteins according to their biological pathways. As selective growth advantage can occur only through the “target pathways”, we have considered only the proteins included in the “target pathways” for our analysis.

### Differential network analysis

For each cancer, in order to identify whether the network structures of the “target pathways” have changed from the “complete remission” group to the “progression” group, we have performed differential network analysis [5] using the R package *dna* [6] . This differential network analysis for each pathway is conducted based on connectivity scores between the proteins in these “target pathways”. The difference in connectivity between the two groups (“progression” versus “complete remission”) is computed mathematically, using the following statistic:

1 $$ \varDelta \left(\mathcal{F}\right)=\frac{1}{k\left(k-1\right)}{\displaystyle \sum_{p\ne {p}^{\prime}\in \mathcal{F}}}\left|{s}_{p{p}^{\prime}}^{pr}-{s}_{p{p}^{\prime}}^{cr}\right| $$

where $\mathcal{F}$ denotes the set of proteins present in a “target pathway” and $k$ denotes the number of proteins in $\mathcal{F}$. Here *s*_*pp* '_^*pr*^ and *s*_*pp* '_^*cr*^ are the connectivity scores between the proteins *p* and *p* ' in the “progression” and “complete remission” groups, respectively. For our analysis, the connectivity score of a protein pair in a network is taken to be the Pearson’s correlation coefficient of the expression values of the two proteins in the corresponding sample data. A permutation test is then carried out using the test statistic ∆(*F*) as follows: let *p* denote the number of proteins in the sample (132 in this case). Let *N*_*pr*_ and *N*_*cr*_ denote the number of patients in the “progression” group and in the “complete remission” group, respectively. The expression profiles of the patients in the two groups are given in the matrix form of orders *N*_*pr*_ × *p* and *N*_*cr*_ × *p*, respectively. Now, a matrix *E* is constructed by merging the rows of the two profiles, i.e., *E* is of order (*N*_*pr*_ + *N*_*cr*_) × *p.* Then, the rows of *E* are randomly permuted and the first *N*_*pr*_ patients are considered as one group and the remaining *N*_*cr*_ patients as another group. For the *i* ^*th*^ permutation, the connectivity scores *s*_*pp* '_^*pr*,*i*^ and *s*_*pp* '_^*cr*,*i*^ between the proteins *p* and *p* ' are then computed using the expression profiles of the permuted data. Thus, the *i* ^*th*^ permuted test statistic ∆(*F,i*) is given by

2 $$ \varDelta \left(\mathcal{F},i\right)=\frac{1}{k\left(k-1\right)}{\displaystyle \sum_{p\ne {p}^{\prime}\in \mathcal{F}}}\left|{s}_{p{p}^{\prime}}^{pr,i}-{s}_{p{p}^{\prime}}^{cr,i}\right| $$

This process is repeated 1000 times and the observed level of significance (*p*-value) is obtained using

3 $$ pval\left(\mathrm{\mathcal{F}}\right)=\frac{1}{1000}{\displaystyle \sum_{i=1}^{1000}}I\left(\Delta \left(\mathrm{\mathcal{F}},i\right)\ge \Delta \left(\mathrm{\mathcal{F}}\right)\right) $$

Next, we have constructed graphical networks for those “target pathways” for which the p-values are significant, so that we get an idea about the network structures in each of the two groups. The graphical networks are constructed by connecting each pair of proteins for which the connectivity score exceeds a threshold.

In addition to testing the overall pathway significance for each cancer, we have also tested whether the connectivity of each single protein has changed between the two groups (“progression” vs “complete remission”) using the following statistic:

4 $$ d(p)=\frac{1}{f-1}{\displaystyle \sum_{p^{\prime}\in \mathcal{G},\kern1em {p}^{\prime}\ne p}}\left|{s}_{p{p}^{\prime}}^{pr}-{s}_{p{p}^{\prime}}^{cr}\right| $$

Where $\mathcal{G}$ denotes the set of all proteins and *f* is the number of proteins in $\mathcal{G}$. Once again, a permutation test is carried out for each protein, in the way described before. The *p*-value corresponding to each protein is obtained using Equation 3, with ∆(*F*) and ∆(*F, i*) replaced by *d*(*p*) and *d*(*p, i*) respectively, where *d*(*p, i*) is given by

5 $$ d\left(p,i\right)=\frac{1}{f-1}{\displaystyle \sum_{p^{\prime}\in \mathcal{G},\kern1em {p}^{\prime}\ne p}}\left|{s}_{p{p}^{\prime}}^{pr,i}-{s}_{p{p}^{\prime}}^{cr,i}\right| $$

### Rank aggregation

The *p*-values, obtained using the test statistic given in Equation 1, are used to obtain ranked lists of the “target pathways” for each cancer type. Here, ranking is done in such a way that the “target pathway” with the lowest p-value gets rank 1, the next one gets rank 2 and so on. Since, these ranked lists vary according to the cancer type; we need to aggregate them in a meaningful way to get an overall ranked list which would then rank the pathways by their global order of importance. In other words, this overall ranked list may provide us with the most important “target pathways” in all the three cancers. In order to get this overall ranked list, we have used the R package *RankAggreg* [12, 13] based on the Cross-entropy Monte Carlo algorithm [25]. In the framework of optimization problem, *RankAggreg* [12, 13] minimizes an objective function, so that a final ordered list is obtained which is close to all the individual ordered lists, simultaneously. The objective function is defined as follows

6 $$ \mathrm{O}(x)={\displaystyle \sum_{i=1}^m}{w}_id\left(x,{Y}_i\right) $$

Here, *Y*_*i*_ is the *i* ^*th*^ ordered list, *x* is the proposed ordered list, *d* is a distance measure and *w*_*i*_ denotes the weight associated with the ordered list *Y*_*i*_. The aim here is to find x* for which the objective function O(*x*), given in Equation 6, is minimum. In other words,

7 $$ {x}^{*}= argmin{\displaystyle \sum_{i=1}^m}{w}_id\left(x,{Y}_i\right) $$

For our analysis, we have considered the *p*-values, obtained from Equation 3, as our weights (*w*_*i*_). Here, *Y*_*i*_ is the *i* ^*th*^ ordered list of “target pathways”. As a distance measure, we have considered weighted Spearman’s footrule distance [26]. A brief description of the algorithm of the weighted Spearman’s footrule distance, used for our purpose, is as follows: Let *p*_*i*_(1); *p*_*i*_(2); … ; *p*(5) be the p-values (in ascending order) associated with the *i* ^*th*^ ordered list, *Y*_*i*_. Let *r*_*A*_(*Y*_*i*_) and *r*_*A*_(*x*) denote the ranks of the “target pathway” A in the *i* ^*th*^ ordered list *Y*_*i*_ and the proposed ordered list *x*, respectively. Then, the weighted Spearman’s footrule distance is given by

8 $$ S\left(x,\ {Y}_i\right)={\displaystyle \sum_{t\epsilon {Y}_i\cup x}}\left|p\left({r}_t\left({Y}_i\right)\right)-p\left({r}_t(x)\right)\right|\left.\times \right|\left.{r}_t\left({Y}_i\right)-{r}_t(x)\right| $$

For our second analysis at the individual protein level, the *p*-values, obtained using the test statistic given in Equation 4, are used to rank the set of 132 individual proteins. An overall ranked list of these proteins is obtained using the R package *RankAggreg* [12, 13] in a similar way.

## Reviewers’ comments

The authors thank the reviewers for their comments. When possible, the manuscript has been modified according to their recommendations.

### Reviewer 1 (Joaquin Dopazo)

The manuscript “Exploring the Importance of Cancer Pathways by Meta-Analysis of Differential Protein Expression Networks in Three Different Cancers by Sikdar et al., presents an elegant comparative analysis of the differences between good and bad prognostic in different cancers in terms of pathway activity instead of using the conventional gene-based perspective.

There are, however, several points that need some clarification for the proper understanding of the manuscript. It is not clear for me how the subnetworks (pathways) have been defined? I am not sure if they used the g: profiler for this purpose (BTW, what is the justification of using the g: profiler? because it is not among the most cited tools for functional analysis). Or they used the connectivity values (taken from coexpressions) to define the edges? If so, what threshold is used to define a connection?

The results are interesting because they show how, taking into account the proteins within the context of the network of interactions linking them, is superior to the conventional one-gene-at-a- time approaches. However, I think this fact, which is one of the main contributions of the paper, is not sufficiently reflected in the manuscript. It would be nice if they can include a table or any type of comparative where they can prove that they can find richer results analysing these proteins within the pathways than analysing them alone.

Additionally, it is worth mentioning, within the context of network analysis, new approaches based on the 3D solved interactome, such as Porta-Pardo et al., 2015, PLoS Comp Biol http://journals.plos.org/ploscompbiol/article?id=10.1371/journal.pcbi.1004518.

Author’s response: *We have used g: profiler for grouping the proteins functionally because it can handle much larger collection of features, for example, genes, proteins,* etc.*, compared to DAVID. We have also used DAVID with KEGG database and got three “target pathways” in common with g: profiler and more than 93% of the proteins were common between the two results (details not shown). In addition, we have tried other pathway systems, namely, PANTHER and BIOCARTA and got, respectively, four and five “target pathways” in common with our results (details not shown).*

*To answer the second part of the comment, we have used connectivity scores with a threshold value of 0.7 to define the edges.*

*We have conducted tests of differential connectivity of each individual protein between the two groups (progression vs complete remission) and considered the top fifty differentially connected proteins for each of the three “target pathways” which were then rank aggregated. These results are shown in Fig.* 3 *. These results are contrasted with the results from pathway level analysis (Table* 2 *) in the* *Discussion* *section of the revised paper. We also note that our pathway level analysis uses a natural normalization with respect to the size of the underlying pathway.*

*Thank you for the reference. It has been cited in the revised paper (see the* *Discussion* *section).*

### Reviewer 2 (Samiran Ghosh)

It is a nice conference article describing meta-analysis. The authors used rank based approach. My only suggestion if they considered random effect based meta -analysis approach also. Overall nice paper.

Author’s response: *We prefer our nonparametric approach since reliable parametric models incorporating random effects terms are not available for the current problem. In addition, the purpose of our "meta-analysis" is somewhat different from the more common use of the term.*
